# TRACE: a new protocol for ultrasound examination during out-of-hospital cardiac arrest

**DOI:** 10.1186/cc14496

**Published:** 2015-03-16

**Authors:** J Callerova, R Skulec, J Knor, V Cerny

**Affiliations:** 1 Emergency Medical Service of Central Bohemian Region, Beroun, Czech Republic; 2 Charles University in Prague, University Hospital Hradec Kralove, Czech Republic; 3 J.E. Purkinje University, Masaryk Hospital, Usti nad Labem, Czech Republic

## Introduction

Implementation of point-of-care ultrasound examination into cardiopulmonary resuscitation (CPR) may increase diagnostic accuracy for determining the cause of cardiac arrest. However, current protocols either do not reflect all causes detectable by ultrasound or are too complicated for prehospital use. Thus, we decided to construct and validate a new protocol TRACE (ThoRacic and Abdominal sonography in Cardiac arrEst) for ultrasound examination during out-of-hospital cardiac arrest (OHCA).

## Methods

We designed a new protocol for ultrasound examination during OHCA to increase the success rate for the establishment of OHCA cause. The subcostal view was performed during planned rhythm check to assess the presence of cardiac tamponade and size of the right and left ventricle and inferior caval vein. Thereafter, during ongoing cardiac compressions, Morrison's pouch and right pleural space were investigated to exclude intraperitoneal and inrapleural free fluid. The same procedure was applied on the left side of the body. Finally, the anterior thoracic view was done to exclude pneumothorax. Working diagnosis was compared with the final in-hospital diagnosis or autopsy.

## Results

We examined 40 consecutive OHCA patients. Correct cause of OHCA during CPR was recognised in 38 patients (95%). Leading causes were acute coronary syndrome (55.0%), pulmonary embolism (15.0%) and complication of chronic heart failure (10.0%). Incorrect recognition was performed in one patient with respiratory cause, originally considered as pulmonary embolism, and in another with pulmonary embolism, considered as respiratory cause. One rhythm check was sufficient to perform TRACE in 31 patients, in the other two interruptions of cardiac compressions were required. Return of spontaneous circulation was achieved in 15 (37.5%) patients, favourable neurological outcome at hospital discharge in eight (20%) patients. Specific therapy to affect the cause of OHCA was applied during OHCA in 12 (30%) patients.

## Conclusion

Implementation of the TRACE protocol to the CPR process was feasible, required minimal interruption of cardiac compressions and resulted in a high recognition rate for the cause of OHCA.

